# Pathologic complete response and survival according to germline *BRCA1/2* status in triple-negative breast cancer

**DOI:** 10.1007/s12282-026-01914-8

**Published:** 2026-09-10

**Authors:** Paulina Gebhart, Florian Heinzl, Chiara Kahl, Alev Bari, Georg Langs, Daniela Muhr, Christian F. Singer, Yen Y. Tan

**Affiliations:** 1 Department of Obstetrics and Gynecology, Comprehensive Cancer Center, Medical University of Vienna, Spitalgasse 23, 1090 Vienna, Austria; 2 Computational Imaging Research Lab, Department of Biomedical Imaging and Image-Guided Therapy, Medical University of Vienna, Spitalgasse 23, 1090 Vienna, Austria; 3 Comprehensive Center for Artificial Intelligence in Medicine, Medical University of Vienna, Spitalgasse 23, 1090 Vienna, Austria

**Keywords:** *BRCA1/2*, Triple-negative breast cancer, Neoadjuvant chemotherapy, Pathologic complete response, Survival

## Abstract

**Background:**

Germline *BRCA1*/*2* pathogenic variants (g*BRCA*-PV) are associated with chemosensitivity in triple-negative breast cancer (TNBC), but whether higher pathologic complete response (pCR) rates translate into improved long-term outcomes remains uncertain. We assessed associations of g*BRCA*-PV status with pCR, recurrence-free survival (RFS) and breast cancer-specific survival (BCSS) in a real-world TNBC cohort treated with neoadjuvant chemotherapy (NACT).

**Methods:**

We retrospectively analyzed 293 patients with stage I-III TNBC diagnosed between 2007 and 2023 who were treated with NACT and underwent germline *BRCA1*/*2* testing at a single tertiary center. Multivariable logistic and Cox regression models assessed pCR, RFS and BCSS.

**Results:**

Of 293 patients, 56 (19.1%) carried g*BRCA*-PV. The overall pCR rate was 49.5% and was higher in carriers than noncarriers (73.2% vs. 43.9%, p<0.001). After adjustment for age at diagnosis, clinical stage, NACT regimen, noncarriers had lower odds of pCR (OR 0.36, 95% CI 0.18–0.70, p=0.004). In Cox models, associations between g*BRCA*-PV status and RFS or BCSS did not reach statistical significance, either with or without adjustment for pCR, and estimates were imprecise. pCR was strongly associated with improved RFS (HR 0.24) and BCSS (HR 0.20) (both p<0.001).

**Conclusions:**

g*BRCA-*PV carriers were more likely to achieve pCR after NACT, whereas associations with RFS or BCSS remained uncertain. The limited number of events and wide confidence intervals preclude firm conclusions regarding survival differences. Larger cohorts with longer follow-up are needed to clarify the relationship between g*BRCA* status, treatment response and long-term outcomes.

**Supplementary Information:**

The online version contains supplementary material available at https://doi.org/10.1007/s12282-026-01914-8.

## Introduction

Triple negative breast cancer (TNBC) accounts for approximately 15–20% of all breast carcinomas and is associated with a poor prognosis [1]. Defined by the absence of estrogen receptor (ER), progesterone receptor (PR) and human epidermal growth factor receptor 2 (HER2) expression [2], TNBC patients do not benefit from endocrine or HER2-targeted treatments. While novel therapies have recently become available for HER2-low disease in the metastatic setting [3], these targeted options remain unavailable for early-stage TNBC. As a result, systemic treatment relies primarily on a chemotherapy-based backbone, particularly in the neoadjuvant setting, to facilitate surgical resection and assess tumor chemosensitivity [4–6]. The recent incorporation of immune checkpoint inhibitors such as pembrolizumab has further improved outcomes and is now routinely included as part of standard neoadjuvant chemotherapy for early-stage TNBC [7].

Achieving pathologic complete response (pCR) after neoadjuvant chemotherapy (NACT) is associated with significantly better event-free and overall survival, particularly for HER2+ and TNBC [1, 7–10]. A recent meta-analysis of real-world data from 22 retrospective studies with more than 12,000 BC patients found that pCR following NACT was significantly associated with a 30% improvement in overall survival (OS) and a 29% improvement in disease-free survival (DFS) [11]. Among TNBC patients, pCR was achieved more frequently and correlated with a 51% improvement in DFS, highlighting its prognostic relevance in this population.

Approximately 15% of TNBC patients harbor germline *BRCA1* or *BRCA2* pathogenic variants (hereafter referred to as g*BRCA-*PV), which impair homologous recombination leading to DNA repair deficiency and genomic instability [12]. These individuals have increased lifetime risk for breast and ovarian cancers and tend to be diagnosed earlier in life [13–15]. Importantly, g*BRCA*-PV tumors exhibit increased sensitivity to DNA-damaging agents, such as platinum-based chemotherapy and poly (ADP-ribose) polymerase inhibitors (PARPi) [16, 17]. While a phase III trial demonstrated improved survival with adjuvant PARPi in g*BRCA-*PV BC patients [18–20], the role of platinum-based NACT in improving long-term outcomes remains uncertain [16, 21]. Several studies have reported that g*BRCA*-PV carriers with TNBC are more likely to achieve pCR and may experience improved outcomes when treated with carboplatin-containing NACT [22–24]. However, the extent to which g*BRCA-*PV status modifies response to chemoimmunotherapy remains unclear.

Despite the well-established association between g*BRCA*-PV status and chemosensitivity, it remains uncertain whether this translates into improved long-term outcome in TNBC, particularly in real-world cohorts treated across an evolving neoadjuvant era [13, 22, 25–27]. We therefore evaluated the association of g*BRCA*-PV status with pCR, recurrence-free survival (RFS) and breast cancer-specific survival (BCSS) in a real-world TNBC cohort treated with NACT, and explored whether different treatment regimens modified these associations.

## Materials and methods

### Study design and patient population

We conducted a retrospective cohort study of patients with TNBC diagnosed between 1 January 2007 and 31 December 2023 at the Department of Obstetrics and Gynecology, Medical University of Vienna. Patients were identified from the department and cross-linkage was performed to identify patients who were tested for breast and ovarian cancer predisposition genes via the hereditary cancer registry (ATHENA).

A total of 1648 patients were screened. Eligible patients met the following criteria: (i) stage I-III disease, (ii) TNBC defined as estrogen receptor (ER) <1%, progesterone receptor (PR) <1%, and HER2-negative status (immunohistochemistry 0, 1+, or 2+ with negative fluorescence in situ hybridization), (iii) receipt of NACT, and (iv) underwent g*BRCA1/2* or multigene panel testing with results available.

Patients were excluded if they did not receive or complete NACT, were pregnant at diagnosis, had metastatic or progressive disease during NACT, were treated outside the study center without sufficiently detailed treatment and pathological response data, or carried a variant of uncertain significance (VUS) or a pathogenic variant in a non-*BRCA* gene. Patients with a known prior or synchronous ipsilateral or contralateral breast cancer that did not meet the TNBC eligibility criteria were also excluded. The hereditary cancer registry also includes patients referred from external centers across Austria for genetic counseling and testing. Many such patients lacked sufficiently detailed treatment and pathological response data for inclusion.

### Clinical, pathological and data sources

All patients underwent standard diagnostic workup, including mammography, ultrasound and/or magnetic resonance imaging (MRI). Staging investigations to exclude metastatic disease included computed tomography (CT) and/or bone scintigraphy, according to institutional practice. Diagnosis of invasive BC was confirmed by core needle biopsy, with histopathological evaluation by dedicated breast pathologists following standardized protocols. Clinicopathological characteristics and treatment data were extracted from electronic medical records. Genetic data were extracted from the ATHENA registry, and survival outcomes were obtained via linkage with Statistics Austria.

### Molecular genetic testing

Germline genetic testing was performed as part of routine clinical care at the Center for Familial Breast and Ovarian Cancer, Medical University of Vienna. Genomic DNA was extracted from peripheral blood and quantified using Qubit (Life Technologies, Carlsbad, CA, USA) and/or a NanoDrop (Thermo Fisher Scientific Inc., Waltham, MA, USA). Before 2015, testing included denaturing high-performance liquid chromatography, Sanger sequencing and/or multiplex ligation-dependent probe amplification (MLPA) for large rearrangements. From 2015 onwards, next-generation sequencing (NGS) was implemented using Illumina TruSight Cancer or Sophia Genetics HCS panel on MiniSeq or MiSeq Dx platforms (Illumina, San Diego, CA, USA). A panel of 18 cancer predisposition genes was analyzed, including *BRCA1, BRCA2, ATM, BRIP1, BARD1, CHEK2, CDH1, PALB2, RAD51C, RAD51D, MLH1, MSH2, MSH6, PMS2, EPCAM, TP53, PTEN*, and *STK11*. Variants were classified according to ACMG/AMP guidelines [28] using SophiaGenetics DDM® and/or Varsome Clinical [29]. Only patients with pathogenic or likely pathogenic variants (class 4 and 5) in *BRCA1/2* were classified as g*BRCA*-PV carriers, whereas those with class 1 or class 2 variants were considered noncarriers. Individuals with variants of uncertain significance (VUS) and PVs in non-*BRCA* genes were excluded.

### Treatment regimens

NACT regimens were administered based on evolving institutional standards over the study period. Most patients received anthracycline- and taxane-based chemotherapy, with or without the addition of carboplatin. From October 2020 onwards, immune checkpoint inhibition such as pembrolizumab was also included for selected patients. For analytical purposes, treatments were categorized into three groups: (1) no platinum, (2) platinum-based and (3) platinum plus pembrolizumab. All patients subsequently underwent definitive breast surgery, followed by adjuvant therapy as clinically indicated based on treatment response, tumor stage, and/or genetic predisposition.

### Endpoints

The primary endpoint was pathologic complete response (pCR), defined as the absence of invasive cancer in both the breast and axillary lymph nodes following neoadjuvant chemotherapy (ypT0/is ypN0), irrespective of the presence of ductal carcinoma in situ. Recurrence-free survival (RFS) was defined as the time from diagnostic core biopsy to the first documented locoregional or distant recurrence. Locoregional recurrence was defined as recurrence in the ipsilateral breast or chest wall and/or regional lymph nodes, whereas distant recurrence was defined as metastatic disease outside these sites. For descriptive reporting, recurrence patterns were classified as locoregional only, distant only, concurrent locoregional and distant recurrence, or recurrence with unavailable site information. Patients with involvement of multiple distant organ sites contributed to each applicable organ-site category. Recurrence was ascertained from routine clinical documentation, including pathology and imaging reports. Where available, recurrent disease was pathologically confirmed. All locoregional recurrences were pathologically confirmed, whereas brain metastases were diagnosed radiologically. Other distant recurrences were classified according to the diagnosis documented in routine clinical records. ER, PR, and HER2 reassessment at recurrence was not systematically documented. New contralateral breast cancers and *BRCA*-associated non-breast malignancies arising during follow-up were not systematically collected as separate variables.

Patients without recurrence were censored at the date of last follow-up, and those who died without a documented recurrence were censored at the date of death. Breast cancer-specific survival (BCSS) was defined as the time from diagnostic biopsy to death attributable to breast cancer. Patients alive at last follow-up or deceased from other causes were censored accordingly.

### Statistical analysis

Baseline characteristics were summarized using descriptive statistics. Group comparisons were performed using Chi-square test or Barnard’s exact test for 2×2 contingency tables with small cell counts, and Mann–Whitney U tests for continuous variables.

Multivariable logistic regression was used to identify independent predictors of pCR. Covariates were selected a priori based on clinical relevance and included age at diagnosis, clinical stage, NACT regimen, and g*BRCA* status (carriers vs. noncarriers). Results are presented as odds ratios (ORs) with 95% confidence intervals (CIs). Due to limited numbers of g*BRCA2-*PV carriers, *gBRCA1-PV* and *gBRCA2-PV* carriers were combined for primary analyses.

Survival outcomes (RFS and BCSS) were estimated using the Kaplan–Meier method. Associations with survival were evaluated using Cox proportional hazards regression models. Model 1 included g*BRCA* status, age at diagnosis, clinical stage, NACT regimen, and pCR status as covariates. Because pCR may lie on the pathway between g*BRCA*-PV status and survival, we also fitted Model 2 without pCR to examine how adjustment for treatment response influenced the association with g*BRCA*-PV status. These models were intended to aid interpretation and were not considered a formal mediation analysis. Results are reported as hazard ratios (HRs) with 95% CI. Given the limited number of recurrence events among g*BRCA*-PV carriers, recurrence subtypes and distant metastatic sites were summarized descriptively and were not evaluated in separate multivariable time-to-event models. Median follow-up time was estimated using the reverse Kaplan–Meier method, with time defined from diagnosis to last contact or death.

To address the potential guarantee-time bias related to pCR being established only at surgery, we additionally performed a sensitivity analysis using the date of surgery as the time origin. RFS was calculated from surgery to first documented recurrence and BCSS from surgery to breast cancer-specific death, with censoring as described above. The same covariates as in Model 1 were included. For one patient, only the month and year of surgery were available; the 15th of the month was assigned and sensitivity analyses using the first or last day of that month did not alter the estimates.

Complete-case analysis was applied for multivariable models. No imputation of covariates used in the primary models was performed. All statistical tests were two-sided, with a significance threshold of p<0.05. All analyses were conducted using R (version 4.5.1) with ggplot2 v4.0.0, viridis v0.6.5, Barnard v1.8, survival v3.8.3, and ggsurvfit v1.2.0 packages.

## Results

**Fig. 1:**
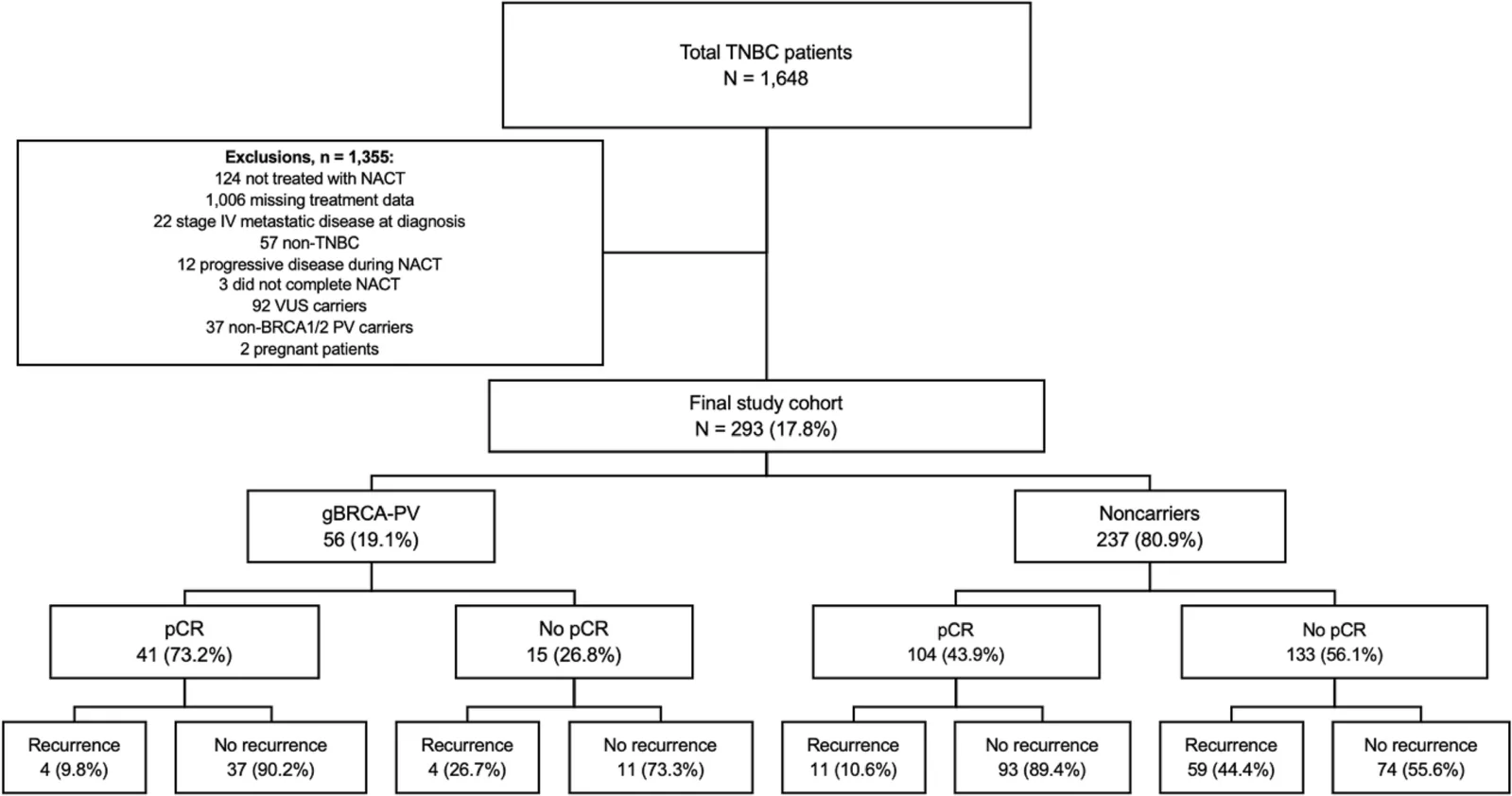
Study cohort inclusion

Of the 1648 TNBC patients identified during the study period, 293 met the eligibility criteria and were included in the final analysis (Fig. 1 and Table 1). Among these, 56 patients (19.1%) carried g*BRCA*-PVs, including 46 g*BRCA1* (82.1%) and 10 g*BRCA2* (17.9%) carriers, whereas 237 patients (80.9%) were classified as noncarriers.

**Table 1:** Cohort characteristics according to germline *BRCA* status

	PV carriers N (%)^a^56 (19.1)		Noncarriers N (%)^a^237 (80.9)		P-value^b^
**Age at diagnosis**					
Median (range) years	41 (26–75)		51 (24–84)		**< 0.001**
**Menopausal status**					
Premenopausal	42 (75.0)		122 (51.5)		**0.001**
Postmenopausal	14 (25.0)		115 (48.5)		
**Body Mass Index (BMI)**					0.579
Underweight	3 (5.4)		5 (2.1)		
Normal weight	27 (48.2)		102 (43.0)		
Overweight	15 (26.8)		70 (29.5)		
Obese	11 (19.6)		48 (20.3)		
Missing^c^	-		12 (5.1)		
**Histologic Subtype**					0.429
IDC	53 (94.6)		222 (93.7)		
ILC	1 (1.8)		1 (0.4)		
Other^d^	2 (3.6)		14 (5.9)		
**Grading**					0.197
1	-		-		
2	3 (5.4)		26 (10.9)		
3	53 (94.6)		208 (87.8)		
Missing^c^	-		3 (1.3)		
**Ki67**					0.136
Median (range)	80 (20–95)		80 (10–100)		
Clinical stage*					**0.013**
I	24	(42.9)	76	(32.1)	
II	32	(57.1)	131	(55.3)	
III	0	(0.0)	30	(12.7)	
Tumor stage*					**0.043**
1	29	(51.8)	94	(39.7)	
2	26	(46.4)	107	(45.2)	
3	1	(1.8)	22	(9.3)	
4	0	(0.0)	14	(5.9)	
**Lymph node metastases**					**0.027**
No	45	(80.4)	154	(65.0)	
Yes	11	(19.6)	83	(35.0)	
**Surgery**					**< 0.001**
Breast-conserving surgery	5	(8.9)	194	(81.9)	
Mastectomy	3	(5.4)	40	(16.9)	
rrBM	48	(85.7)	3	(1.3)	
**Axilla dissection**					0.065
No	44	(78.6)	156	(65.8)	
Yes	12	(21.4)	81	(34.2)	
**Radiotherapy**					**< 0.001**
No	38	(69.1)	16	(6.8)	
Yes	17	(30.9)	219	(92.4)	
Missing^c^	1	(1.8)	2	(0.8)	
**NACT regimen**					**< 0.001**
No platinum	13	(23.2)	115	(48.5)	
Platinum-based	36	(64.3)	79	(33.3)	
Platinum-based with pembrolizumab	7	(12.5)	43	(18.1)	
**pCR**					**< 0.001**
No	15	(26.8)	133	(56.1)	
Yes	41	(73.2)	104	(43.9)	
**Adjuvant therapy** (non-pCR patients only)					**< 0.001**
No adjuvant therapy	8	(53.3)	57	(42.9)	
Capecitabine	2	(13.3)	46	(34.6)	
Pembrolizumab	1	(6.7)	15	(11.3)	
Olaparib	3	(20.0)	0	(0)	
Other Chemotherapy	1	(6.7)	15	(11.3)	
**Death (breast cancer-specific)**					**0.021**
No	53	(94.6)	195	(82.3)	
Yes	3	(5.4)	42	(17.7)	
**Recurrence**^e^					**0.021**
No	48	(85.7)	167	(70.5)	
Any recurrence	8	(14.3)	70	(29.5)	
Locoregional only	1	(1.8)	21	(8.9)	
Distant only	3	(5.4)	34	(14.3)	
Both locoregional and distant recurrence	2	(3.6)	5	(2.1)	
Recurrence site unavailable^f^	2	(3.6)	10	(4.2)	

g*BRCA1/2* germline *BRCA1* or germline *BRCA2*; PV pathogenic variant; NACT neoadjuvant chemotherapy; rrBM risk-reducing bilateral mastectomy, also includes nipple-sparing bilateral mastectomy

^a^ Column percentage presented

^b^ P-values were calculated using the Chi-square test, Barnard’s exact test or Mann–Whitney U test, as appropriate

^c^ Excluded from analyses of the corresponding variable

^d^ Invasive anaplastic (n=3), invasive micropapillary (n=1), invasive medullary (n=4), invasive apocrine (n=3), invasive metaplastic (n=5)

^e^ Locoregional recurrence includes recurrence in the ipsilateral breast/chest wall and/or regional lymph nodes. Distant recurrence includes metastatic disease outside locoregional sites

^f^ Site information was unavailable in the clinical record

^*^ According to the 8th Edition of the Union for International Cancer Control (UICC) Tumor, Node, Metastasis (TNM) classification

Bold values indicate statistical significance at p < 0.05.

Compared with noncarriers, g*BRCA*-PV carriers were significantly younger at diagnosis (p<0.001) and more likely to be premenopausal (p=0.001). They also presented with less advanced disease, including lower tumor stage (p=0.043), fewer lymph node metastases (p=0.027), and earlier clinical stage (p=0.013). In addition, g*BRCA-*PV carriers were more frequently treated with carboplatin-based NACT, whereas noncarriers were more likely to be treated with non-carboplatin regimens (p<0.001). The distribution of patients, g*BRCA*-PV status, neoadjuvant treatment regimens, and pCR rates according to diagnosis period is presented in Supplementary Table S1. Treatment patterns changed over time, with increasing use of platinum-based chemotherapy and the introduction of pembrolizumab-containing regimens during the later study period.

### Predictors of pathologic complete response (pCR)

Overall, 145 of 293 patients (49.5%) achieved pCR following NACT. Among the 148 patients with residual disease, 65 (43.9%) did not receive adjuvant treatment. Among patients with residual disease, three g*BRCA*-PV carriers received adjuvant olaparib but no noncarriers received a PARP inhibitor. The distribution of post-neoadjuvant systemic therapies is presented in Table 1. In the multivariable logistic regression analysis, g*BRCA* status remained independently associated with pCR after adjusting for age at diagnosis, clinical stage, and treatment regimen (Table 2). Because noncarrier status was coded relative to carrier status, the odds ratio below 1.0 indicates a lower likelihood of pCR in noncarriers, and conversely, a higher likelihood of pCR in g*BRCA*-PV carriers (OR for noncarriers vs. carriers 0.36, 95% CI 0.18–0.70, p=0.004). Younger age was also independently associated with pCR (OR 0.98, 95% CI 0.956–1.000, p=0.036), and patients with stage I disease had significantly higher odds of pCR than those with stage III disease (OR 2.49, 95% CI 1.03–6.44, p=0.049). By contrast, NACT regimen was not independently associated with pCR, although a non-significant trend was observed for platinum plus pembrolizumab compared with non-platinum treatment (OR 1.76, 95% CI 0.89–3.52, p=0.105).

**Table 2:** Multivariable logistic regression model for pCR

**Variables**	**OR**	**95% CI**	**p-value**^a^
**Age**	0.98	0.956–1.00	**0.036**
**g*BRCA* status**			
Noncarrier	0.36	0.18–0.70	**0.004**
Carrier	1.00		
**Clinical stage**^**b**^			
I	2.49	1.03–6.44	**0.049**
II	2.01	0.87–4.99	0.113
III	1.00		
**NACT regimen**			
Platinum-based with pembrolizumab	1.76	0.89–3.52	0.105
Platinum-based	1.02	0.58–1.76	0.953
Non-platinum	1.00		

^a^ P-value derived from the multivariable logistic regression model

^*b*^* According to the 8th Edition of the Union for International Cancer Control (UICC) Tumor, Node, Metastasis (TNM) classification*

Bold values indicate statistical significance at p < 0.05

### Predictors of recurrence-free survival (RFS)

In the Cox regression analyses, the estimated association between g*BRCA*-PV status and RFS differed modestly according to whether pCR was included (Table 3). In Model 1 with pCR included, noncarriers had a higher estimated risk of recurrence than g*BRCA*-PV carriers, although the association was not statistically significant (HR 1.73, 95% CI 0.79–3.80, p=0.168). In Model 2, which excluded pCR, the corresponding estimate was HR 2.04 (95% CI 0.93–4.46, p=0.075). The modest attenuation after adjustment for pCR is consistent with treatment response potentially accounting for part of the association between g*BRCA*-PV status and recurrence.

**Table 3:** Cox proportional hazards models for recurrence-free survival and breast cancer-specific survival, with and without adjustment for pathologic complete response^a^

Variable	Model 1: with pCR			Model 2: without pCR		
	**HR**	**95% CI**	**p-value**	**HR**	**95% CI**	**p-value**
**Recurrence-free survival**						
Age at diagnosis	0.99	0.97–1.01	0.373	1.00	0.98–1.02	0.932
g*BRCA* status						
Noncarrier	1.73	0.79–3.80	0.168	2.04	0.93–4.46	0.075
Carrier	1.00			1.00		
Clinical stage*						
I	0.27	0.13–0.56	**< 0.001**	0.23	0.11–0.47	**< 0.001**
II	0.59	0.33–1.06	0.075	0.51	0.29–0.90	**0.019**
III	1.00			1.00		
NACT regimen						
Platinum-based with pembrolizumab	2.29	1.13–4.67	**0.022**	1.83	0.90–3.71	0.093
Platinum-based	1.48	0.89–2.47	0.133	1.44	0.87–2.39	0.158
Non-platinum	1.00			1.00		
pCR						
Yes	0.24	0.14–0.43	**< 0.001**	**-**	**-**	**-**
No	1.00			**-**		
**Breast cancer-specific survival**						
Age at diagnosis	1.02	0.99–1.04	0.227	1.03	1.00–1.05	0.060
g*BRCA* status						
Noncarrier	2.34	0.67–8.13	0.183	2.30	0.67–7.96	0.187
Carrier	1.00			1.00		
Clinical stage*						
I	0.12	0.04–0.38	**< 0.001**	0.10	0.03–0.30	**< 0.001**
II	0.57	0.28–1.15	0.117	0.44	0.22–0.86	**0.017**
III	1.00			1.00		
NACT regimen						
Platinum-based with pembrolizumab	0.75	0.17–3.28	0.705	0.65	0.15–2.83	0.565
Platinum-based	1.32	0.68–2.59	0.412	1.20	0.63–2.31	0.581
Non-platinum	1.00			1.00		
pCR						
Yes	0.20	0.08–0.48	**< 0.001**	-	-	-
No	1.00			-		

^a^ Model 1 included age at diagnosis, g*BRCA* status, clinical stage, and NACT regimen and pCR status. Model 2 excluded pCR status. Analyses included all 293 patients. Hazard ratios (HR) are presented with 95% confidence intervals (95%CI). Carrier status, clinical stage III, non-platinum NACT, and no pCR were used as reference categories, as applicable

^*^ According to the 8th Edition of the Union for International Cancer Control (UICC) Tumor, Node, Metastasis (TNM) classification

Bold values indicate statistical significance at p < 0.05

**Fig. 2:**
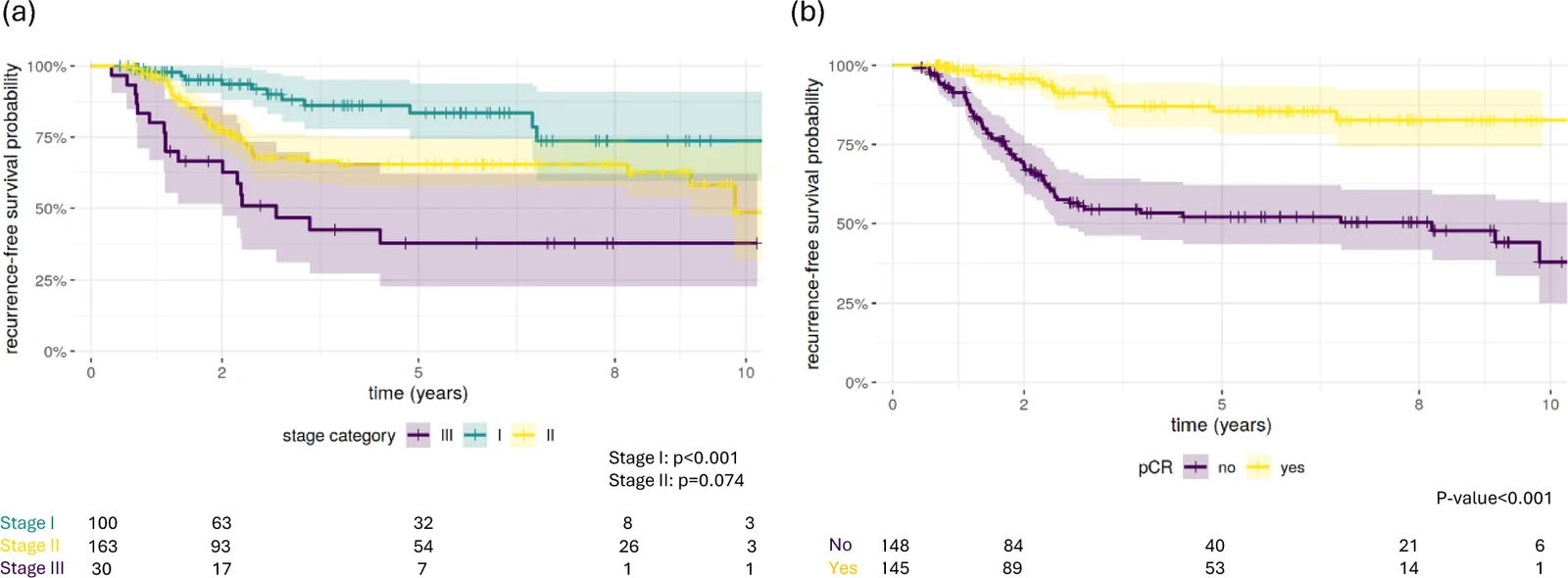
Recurrence-free survival by clinical stage and pCR status

In Model 1, patients with stage I disease had a lower risk of recurrence than those with stage III disease (HR 0.27, 95% CI 0.13–0.56, p<0.001) (Fig. 2a). pCR was strongly associated with improved RFS (HR 0.24, 95% CI 0.14–0.43, p<0.001) (Fig. 2b). Patients treated with platinum plus pembrolizumab had a higher estimated risk of recurrence than those treated without platinum (HR 2.29, 95% CI 1.13–4.67, p=0.022), whereas platinum-based chemotherapy without pembrolizumab was not significantly associated with RFS (HR 1.48, 95% CI 0.89–2.47, p=0.133).

### Predictors of breast cancer-specific survival (BCSS)

During a median follow-up of 4.1 years, 45 breast cancer-specific deaths occurred. The estimated association between g*BRCA*-PV status and BCSS was very similar in the two models (Table 3). In Model 1, with pCR included, the HR for noncarriers compared with g*BRCA*-PV carriers was 2.34 (95% CI 0.67–8.13; p=0.183). In Model 2, which excluded pCR, the corresponding HR was 2.30 (95% CI 0.67–7.96; p=0.187), indicating that adjustment for pCR had little influence on the estimated association with BCSS.

**Fig. 3:**
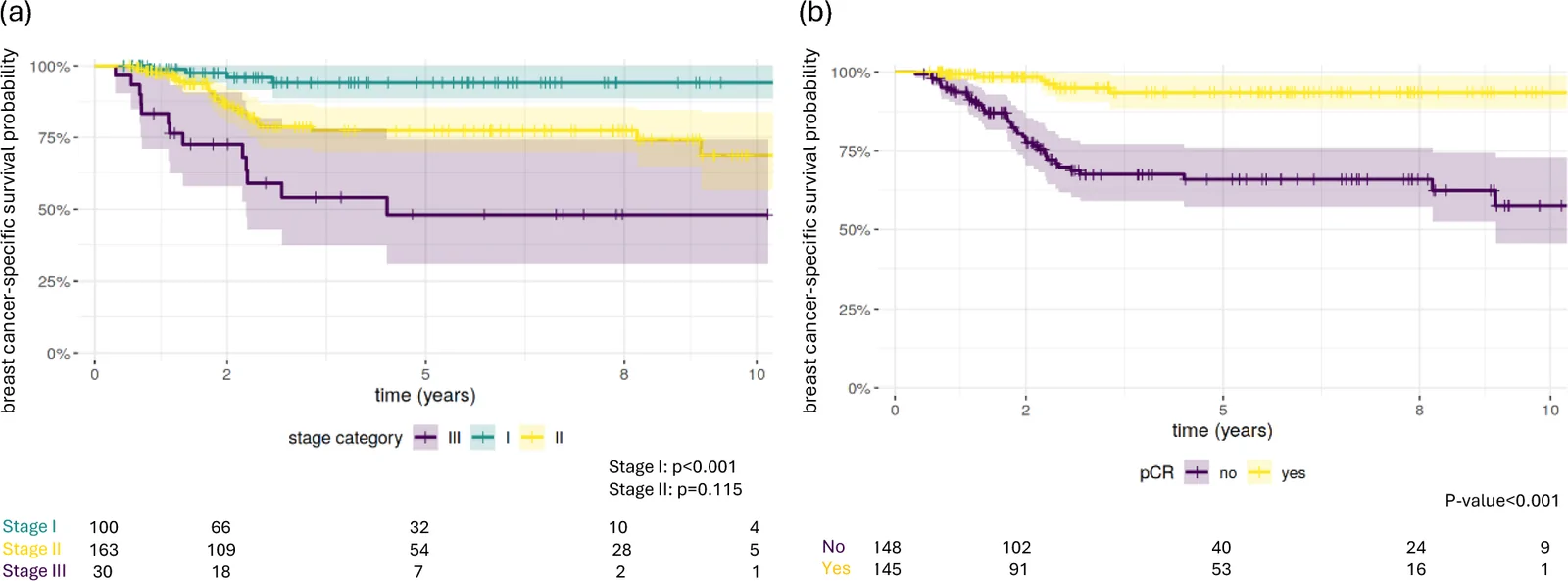
Breast cancer-specific survival by clinical stage and pCR

In Model 1, patients with stage I disease had a lower risk of breast cancer-specific death than those with stage III disease (HR 0.12, 95% CI 0.04–0.38, p<0.001) (Fig. 3a). pCR was strongly associated with improved BCSS (HR 0.20, 95% CI 0.08–0.48, p<0.001) (Fig. 3b). Age at diagnosis and NACT regimen were not significantly associated with BCSS.

Although pCR and early clinical stage were the strongest predictors of favorable BCSS, a subset of patients who achieved pCR still developed recurrence or died from breast cancer. Table 4 further shows case-level detail for the 15 patients who achieved pCR but subsequently developed recurrence. Most of these patients were noncarriers (11/15), and breast cancer-specific death occurred in 6 of the 15 cases, indicating that although uncommon, relapse after pCR can still be clinically consequential. Not all deaths after recurrence were breast cancer-specific, further supporting the use of BCSS as the more appropriate endpoint in this cohort.

**Table 4:** Clinical characteristics of patients who developed recurrence after achieving pCR

Case no	g*BRCA* status	Age at diagnosis	Menopause status	BMI	Type of recurrence	Deceased	Cause of death
76	WT^1^	51	Postmenopause	Overweight	Locoregional	No	
191	WT	48	Premenopause	Normal weight	Locoregional	**Yes**	Breast cancer
256	WT	62	Postmenopause	Normal weight	Lung	**Yes**	Breast cancer
305	WT	45	Premenopause	Overweight	Unknown	**Yes**	Breast cancer
306	*BRCA2*	35	Premenopause	Overweight	Lung	No	
376	WT	47	Premenopause	Normal weight	Lung	**Yes**	Breast cancer
560	*BRCA2*	34	Premenopause	Normal weight	Liver	No	
991	*BRCA2*	75	Postmenopause	Normal weight	Unknown	**Yes**	Breast cancer
1396	WT	47	Premenopause	Obese	Lung, brain, bone	**Yes**	Cardiovascular arrest
1401	*BRCA1*	30	Premenopause	Normal weight	Lung, pancreas	**Yes**	Sepsis
1410	WT	29	Premenopause	Normal weight	Locoregional	No	
1447	WT	39	Premenopause	Normal weight	Skin, liver, lung, bone	**Yes**	Liver failure
1570	WT	57	Postmenopause	Normal weight	Brain, bone	**Yes**	Breast cancer
1603	WT	26	Premenopause	Overweight	Locoregional	No	
1625	WT	40	Premenopause	Normal weight	Locoregional	No	

WT wild-type = noncarrier

### Surgery-based sensitivity analysis

When follow-up was recalculated using the date of surgery as the time origin, pCR remained strongly associated with improved RFS (HR 0.24, 95% CI 0.14–0.43, p<0.001) and BCSS (HR 0.20, 95% CI 0.08–0.48, p<0.001) (Supplementary Table S4). These estimates were essentially unchanged from the diagnosis-origin analyses.

### Patterns of recurrence

Overall, recurrence occurred in 8 of 56 g*BRCA*-PV carriers (14.3%) and 70 of 237 noncarriers (29.5%; p=0.021). Of the 78 patients with recurrence, 65 (83.3%) had pathological confirmation. Recurrence patterns were classified as locoregional only, distant only, concurrent locoregional and distant recurrence, or recurrence with unavailable site information (Table 1). The distribution of documented distant metastatic sites is presented in Supplementary Table S2.

Gene-specific descriptive outcomes differed substantially. Among 46 g*BRCA1*-PV carriers, recurrence occurred in three patients (6.5%) and breast cancer-specific death in one patient (2.2%). In contrast, among 10 g*BRCA2*-PV carriers, recurrence occurred in five patients (50.0%) and breast cancer-specific death in two patients (20.0%) (Supplementary Table S3). Given the small number of g*BRCA2*-PV carriers and outcome events, no formal gene-specific statistical comparisons or survival analyses were performed.

### Exploratory stratified analysis by pCR and g*BRCA* status

To further explore the relationship between pCR, g*BRCA* status, and recurrence, we performed an exploratory stratified analysis. Overall, recurrence was observed in 8/56 carriers (14.3%) and 70/237 noncarriers (29.5%) (p=0.021). Among patients who achieved pCR, recurrence occurred at a similar frequency in carriers and noncarriers (9.8% vs. 10.6%, p=0.884). In contrast, recurrence was more frequent among patients with residual disease, particularly among noncarriers (44.4% vs. 26.7%, p=0.196). Although the pooled carrier group had a lower crude recurrence rate than noncarriers, this difference was driven predominantly by the g*BRCA1*-PV subgroup and should not be interpreted as evidence of uniformly favorable outcomes among g*BRCA*-PV carriers. Given the limited number of events, subgroup analyses should be interpreted cautiously.

## Discussion

In this real-world cohort of patients with TNBC treated with NACT, g*BRCA*-PV carriers were significantly more likely to achieve pCR than noncarriers. The association between g*BRCA*-PV status and long-term outcomes remained uncertain. In Cox models excluding pCR, noncarriers had a higher estimated risk of recurrence than carriers, although the confidence interval was wide and the association did not reach statistical significance. Adjustment for pCR modestly attenuated this estimate, consistent with the possibility that treatment response may account for part of the association between g*BRCA*-PV and recurrence. In contrast, BCSS estimates changed minimally after adjustment for pCR. Given the observational design and the limited number of outcome events, these findings should not be interpreted as a formal mediation analysis. Importantly, the pooled g*BRCA*-PV analysis also obscured differences between *BRCA1* and *BRCA2* carriers. The lower crude recurrence rate among carriers was driven predominantly by g*BRCA1*-PV subgroup, in which 3/46 g*BRCA1*-PV carriers (6.5%) developed recurrence. In contrast, 5/10 g*BRCA2*-PV carriers (50.0%) developed recurrence, while breast cancer-specific death occurred in 1/46 g*BRCA1*-PV carriers (2.2%) versus 2/10 g*BRCA2*-PV carriers (20.0%). Given the small number of g*BRCA2*-PV carriers and outcome events, these findings should be interpreted with caution. The present data therefore cannot establish a true difference in prognosis between g*BRCA1*- and g*BRCA2*-associated TNBC, but they demonstrate that pooled g*BRCA1/2* analyses should not be interpreted as evidence of uniformly favorable prognosis among carriers.

The overall pCR rate in our cohort was 49.5%, with substantially higher rates among *gBRCA*-PV carriers than noncarriers (73.2% vs. 43.9%). This is consistent with prior studies showing enhanced chemosensitivity of *BRCA*-associated TNBC, particularly to DNA-damaging agents such as platinum-based chemotherapy [22–24, 30–32], likely reflecting underlying homologous recombination deficiency and the resulting vulnerability to DNA repair-directed treatment strategies. Importantly, g*BRCA*-PV status remained independently associated with pCR after adjustment for age, clinical stage and NACT regimen.

At the same time, we did not observe an independent association between NACT regimen and pCR, despite the more frequent use of carboplatin-containing regimens in carriers. This is broadly consistent with prospective studies such as GeparSixto [21] and BrighTNess [33], which showed that while carboplatin increased pCR rates overall, the incremental benefit within *BRCA* subgroups was less clear. Taken together, these findings suggest *BRCA*-associated tumors may already exhibit substantial baseline chemosensitivity, such that escalation with platinum may not necessarily produce a proportionate additional gain in pCR. However, our data are not sufficient to support treatment de-escalation in current practice, and this question should be addressed in prospective, response-adapted studies. In the RFS model, pembrolizumab-containing platinum-based NACT was associated with a higher recurrence hazard than non-platinum treatment. This finding should not be interpreted as a treatment effect and may reflect residual confounding by indication, disease severity, and calendar period, particularly given the non-randomized design and shorter follow-up of patients treated in the immunotherapy era. These findings should not be interpreted as evidence that pCR has reduced prognostic value in g*BRCA*-associated TNBC. Rather, they highlight the uncertainty surrounding the relationship between g*BRCA*-PV status, pCR, and long-term outcomes when event numbers are limited. The PARTNER trial reported no improvement in pCR with a specific neoadjuvant olaparib schedule despite exploratory differences in some survival endpoints [34] but the study was small and its survival findings require confirmation. In our cohort, the limited number of events precludes firm conclusions regarding whether the prognostic implications of pCR differ according to g*BRCA*-PV status.

Similar to previous studies [21, 24, 32], pCR emerged as one of the strongest predictors for both RFS and BCSS in our cohort, together with clinical stage at diagnosis. This is consistent with the established role of pCR as a key prognostic marker in TNBC. Because pCR is established only at surgery, we repeated the survival analyses using date of surgery as the time origin. The estimates for pCR remained unchanged, indicating that the strong associations with subsequent RFS and BCSS were not affected by inclusion of the pre-surgical period. However, these analyses remain limited to patients who completed NACT and underwent surgery and do not capture patients with progression during NACT. Importantly, pCR did not eliminate recurrence risk. Exploratory stratified analyses showed that recurrence after pCR was uncommon and occurred at a similar frequency in carriers and noncarriers (9.8% vs. 10.6%, p=0.884). In contrast, recurrence was more frequent among patients with residual disease, particularly among noncarriers (44.4% vs. 26.7%, p=0.196), although this difference did not reach statistical significance. These patterns are compatible with pCR contributing to the pooled difference in recurrence, but the small subgroup sizes and gene-specific heterogeneity preclude firm conclusions. Only eight recurrence events occurred among g*BRCA*-PV carriers during the observation period, substantially limiting statistical power to determine whether higher pCR rates translate into improved long-term outcomes.

The survival models should also be interpreted in light of pCR as a potential intermediate factor between g*BRCA*-PV status and outcome. For RFS, the estimate for noncarriers versus carriers changed from 2.04 without adjustment for pCR to 1.73 after adjustment, suggesting that treatment response may account for part of this association. In contrast, the BCSS estimate changed little. These models are descriptive and were not intended as a formal mediation analysis.

Recurrence after pCR, while infrequent, was not consistently associated with favorable clinical outcomes. Among the 15 patients who developed recurrence after achieving pCR, nine died, including six from breast cancer. Not all deaths after recurrence were breast cancer-specific, further supporting the use of BCSS as the more appropriate endpoint in this cohort. Together, these observations underscore the need for improved post-pCR risk stratification to better identify patients who remain at clinically meaningful risk despite achieving pCR. Future strategies may include circulating tumor DNA (ctDNA) analysis [35, 36], molecular profiling of residual disease, and more individualized surveillance approaches.

From a clinical perspective, these findings have important implications for post-neoadjuvant management. Although pCR remains a key therapeutic goal and prognostic marker, it does not eliminate recurrence risk, including among g*BRCA*-PV carriers. This supports the need for improved post-pCR risk stratification strategies that incorporate dynamic biomarkers, such as circulating tumor DNA, as well as molecular characterization of residual disease and host-tumor interactions. In addition, *BRCA* status alone should not be assumed to confer favorable long-term prognosis, reinforcing the importance of individualized surveillance and adjuvant treatment decisions based on a broader set of risk factors. These considerations are particularly relevant in the context of evolving post-neoadjuvant treatment strategies, including capecitabine [37], PARP inhibitors [18], and continued immunotherapy [38], where optimal patient selection and sequencing remain areas of active investigation [39, 40].

Clinical stage at diagnosis was also an important predictor for pCR, RFS and BCSS. The association between more advanced stage and poorer outcomes is well-established by previous studies [41, 42] and was confirmed in our cohort. Noncarriers were more likely to present with more advanced disease, which may reflect earlier detection and prevention strategies in g*BRCA*-PV carriers, including intensified surveillance and risk-reducing interventions [43, 44]. The younger age of the carrier group may also have contributed to this difference, as older patients generally are at increased risk of delayed diagnosis due to lower screening uptake, later presentation, and age-related barriers to health-seeking behavior [45]. These findings emphasize the continuing importance of early detection and timely treatment, regardless of *BRCA* status.

A key strength of this study is its real-world, molecularly characterized TNBC cohort, with comprehensive germline testing and linkage to national mortality data. Unlike many neoadjuvant *BRCA*-associated TNBC studies that have focused on overall survival, we evaluated BCSS, providing a more direct assessment of the association between g*BRCA*-PV status, tumor biology, pCR, and mortality. This study also has several limitations. First, the retrospective single-center design introduces the potential for selection bias and residual confounding. Because the final cohort was restricted to patients with sufficiently complete treatment and outcome data, it represents a selected population treated at a tertiary referral center with dedicated hereditary cancer expertise. This may limit generalizability to other healthcare settings and referral pathways. Second, the median follow-up of about 4 years captures the period of highest recurrence risk in TNBC, but may be insufficient to fully assess longer-term survival differences, particularly beyond five years. Third, treatment regimens evolved substantially over the extended study period, including increasing use of platinum-containing neoadjuvant regimens, the introduction of pembrolizumab, and the adoption of post-neoadjuvant capecitabine and olaparib. Complete information on treatment completion and post-neoadjuvant systemic therapy was not consistently available. Although only three patients received olaparib, its use may have influenced long-term outcomes among high-risk g*BRCA*-PV carriers treated during the later study period. Residual confounding related to evolving treatment practices and calendar period therefore cannot be excluded. Differences in surgical management may also have influenced locoregional recurrence patterns. Bilateral risk-reducing mastectomy was substantially more frequent among g*BRCA*-PV carriers and may have reduced locoregional recurrence in the group. In addition, ER, PR, and HER2 status at recurrence was not systematically available, limiting assessment of receptor conversion. New contralateral breast cancers and *BRCA*-associated non-breast malignancies arising during follow-up were not systematically captured as separate variables, limiting their independent evaluation. Fourth, the study was not powered to exclude subtle or moderate survival differences between g*BRCA*-PV carriers and noncarriers. Only eight recurrences occurred among carriers, and the number of breast cancer-specific deaths was also small. This limited the precision of the Cox model estimates, as reflected by the wide confidence intervals observed for both RFS and BCSS, and may have reduced model stability. Accordingly, the adjusted survival analyses should be considered exploratory. The possibility of Type II error therefore cannot be excluded, and the absence of statistically significant associations should not be interpreted as evidence of equivalent survival outcomes. Finally, the limited number of events precluded reliable gene-specific survival analyses, separate evaluation of recurrence according to site, and a distinct distant recurrence-free survival analysis. Descriptive outcomes for g*BRCA1*- and g*BRCA2*-PV carriers are therefore presented separately in Supplementary Table S3. Larger multicenter studies with longer follow-up and more complete contemporary treatment data are needed to further clarify the relationship between treatment response and long-term outcomes in *BRCA*-associated TNBC.

## Conclusion

In this retrospective cohort, g*BRCA*-PV carriers with TNBC were more likely to achieve pCR after NACT. Associations between g*BRCA*-PV status and RFS or BCSS were imprecisely estimated and did not reach statistical significance in models with or without adjustment for pCR. Gene-specific descriptive outcomes suggested heterogeneity between g*BRCA1*- and g*BRCA2*-PV carriers, but the limited number of g*BRCA2*-PV carriers precludes firm conclusions. pCR remained strongly associated with both outcomes. Larger studies with more events, longer follow-up, and contemporary treatment data are needed to clarify the relationship between g*BRCA* status, treatment status, and long-term outcomes.

## Supplementary Information

Below is the link to the electronic supplementary material.

Supplementary file1 (DOCX 16 kb)

## Data Availability

The data underlying this study are derived from retrospective clinical records and contain sensitive patient information. Due to institutional policies and ethical/legal restrictions related to patient confidentiality, these data cannot be made publicly available. De-identified data may be made available to qualified researchers upon reasonable request, subject to approval by the appropriate institutional review board and completion of a data use agreement. Requests for access should be directed to the corresponding author.
