# Supplementary material for: Pathologic complete response and survival according to germline BRCA1/2 status in triple-negative breast cancer
Source: Breast Cancer. 2026 Sep 10;33(6):1340–52. doi: 10.1007/s12282-026-01914-8 (PMC13695587; doi:10.1007/s12282-026-01914-8)
Supplement: Supplementary file 1 [file 12282_2026_1914_MOESM1_ESM.docx]

**List of Supplementary Tables**

Supplementary Table S1. Distribution of study participants, neoadjuvant treatment and pathologic response by diagnosis period.

Supplementary Table S2. Distant metastatic site among patients with documented distant recurrence, by gBRCA-PV status.

Supplementary Table S3. Descriptive treatment response and outcomes according to gBRCA1- or gBRCA2-PV status.

Supplementary Table S4. Cox proportional hazards models for recurrence-free survival and breast cancer-specific survival using surgery as the time origin.

Supplementary Table S1. Distribution of study participants, neoadjuvant treatment and pathologic response by diagnosis period.

| **Diagnosis period** | **Cohort distribution^a^**  N (%) | | | **Neoadjuvant treatment^a,b^**  N (%) | | | **Pathologic response^a^** |
| --- | --- | --- | --- | --- | --- | --- | --- |
|  | All patients | g*BRCA*-PV | Noncarriers | No platinum | Platinum-based | Platinum + pembrolizumab | pCR |
| 2007-2013 | 12 (4.1) | 2 (16.7) | 10 (83.3) | 10 (83.3) | 2 (16.7) | 0 (0.0) | 1 (8.3) |
| 2014-2017 | 76 (25.9) | 14 (18.4) | 62 (81.6) | 43 (56.6) | 33 (43.4) | 0 (0.0) | 34 (44.7) |
| 2018-2020 | 84 (28.7) | 13 (15.5) | 71 (84.5) | 42 (50.0) | 41 (48.8) | 1 (1.2) | 42 (50.0) |
| 2021-2023 | 121 (41.3) | 27 (22.3) | 94 (77.7) | 33 (27.3) | 39 (32.2) | 49 (40.5) | 68 (56.2) |
| **Total** | **293 (100.0)** | **56 (19.1)** | **237 (80.9)** | **128 (43.7)** | **115 (39.2)** | **50 (17.1)** | **145 (49.5)** |
| Abbreviations: g*BRCA*-PV, germline *BRCA1/2* pathogenic variant; pCR, pathologic complete response.  ^a^ Column percentage presented for “All patients”; row percentage with “All patients” as denominator presented for all others according to diagnosis-period.  ^b^ Neoadjuvant treatment categories are mutually exclusive. Platinum + pembrolizumab includes pembrolizumab-containing platinum-based regimens. | | | | | | | |

Supplementary Table S2. Distant metastatic site among patients with documented distant recurrence by gBRCA-PV status**^a^**.

|  | **PV carriers**  **N (%)^b^**  **5 (11.4%)** | | **Noncarriers**  **N (%)^b^**  **39 (88.6%)** | | **Total**  **N = 44** |
| --- | --- | --- | --- | --- | --- |
| Bone | 1 | (20.0) | 5 | (12.8) | 6 (13.6) |
| Brain | 1 | (20.0) | 7 | (17.9) | 8 (18.2) |
| Liver/hepatic | 1 | (20.0) | 11 | (28.2) | 12 (27.3) |
| Lung/pleura | 3 | (60.0) | 20 | (51.3) | 23 (52.3) |
| Distant lymph nodes | 0 | (0) | 1 | (2.6) | 1 (2.3) |
| Other | 0 | (0) | 0 | (0) | 0 (0) |
| ^a^ Five patients had more than one documented distant metastatic site  ^b^ Patients with multiple metastatic sites contribute to each applicable category; percentages therefore do not sum to 100%. | | | | | |

Supplementary Table S3. Descriptive treatment response and outcomes according to gBRCA1- or gBRCA2-PV status^a.^

|  | **g*BRCA1-*PV carriers**  **N (%)**  **46 (82.1%)** | **g*BRCA2-*PV carriers**  **N (%)**  **10 (17.9%)** |
| --- | --- | --- |
| Pathologic complete response | 35 (76.1) | 6 (60.0) |
| Residual disease | 11 (23.9) | 4 (40.0) |
| Any recurrence | 3 (6.5) | 5 (50.0) |
| Locoregional only | 0 (0.0) | 1 (10.0) |
| Distant only | 1 (2.2) | 3 (30.0) |
| Concurrent locoregional and distant recurrence | 2 (4.3) | 0 (0.0) |
| Recurrence site unavailable | 0 (0.0) | 1 (10.0) |
| Breast cancer-specific death | 1 (2.2) | 2 (20.0) |
| Abbreviations: g*BRCA1/2* germline *BRCA1/2* pathogenic variant.  ^a^ Data are presented as n (%). Percentages use the gene-specific column total as denominator. Recurrence subcategories are mutually exclusive and sum to the number of patients with any recurrence. No formal comparisons were performed because of the small number of gBRCA2-PV carriers and outcome events. | | |

Supplementary Table S4. Cox proportional hazards models for recurrence-free survival and breast cancer-specific survival using surgery as the time origin.

| **Variable** | **Recurrence-free survival** | | | **Breast cancer-specific survival** | | |
| --- | --- | --- | --- | --- | --- | --- |
|  | **HR** | **95% CI** | **p-value** | **HR** | **95% CI** | **p-value** |
| Age at diagnosis | 0.99 | 0.97-1.01 | 0.339 | 1.02 | 0.99-1.04 | 0.225 |
| g*BRCA* status |  |  |  |  |  |  |
| Noncarrier | 1.71 | 0.78-3.76 | 0.178 | 2.33 | 0.67-8.11 | 0.184 |
| Carrier | 1.00 |  |  | 1.00 |  |  |
| Clinical stage* |  |  |  |  |  |  |
| I | 0.29 | 0.14-0.59 | **< 0.001** | 0.13 | 0.04-0.39 | **< 0.001** |
| II | 0.61 | 0.34-1.10 | 0.099 | 0.58 | 0.28-1.16 | **0.124** |
| III | 1.00 |  |  | 1.00 |  |  |
| NACT regimen |  |  |  |  |  |  |
| Platinum+pembrolizumab | 2.35 | 1.15-4.79 | **0.019** | 0.71 | 0.16-3.06 | 0.642 |
| Platinum-based | 1.49 | 0.90-2.49 | 0.124 | 1.34 | 0.68-2.62 | 0.393 |
| Non-platinum | 1.00 |  |  | 1.00 |  |  |
| pCR |  |  |  |  |  |  |
| Yes | 0.24 | 0.14-0.43 | **< 0.001** | 0.20 | 0.08-0.48 | **< 0.001** |
| No | 1.00 |  |  | 1.00 |  |  |
| ^a^ Time origin was the date of surgery. Models included age at diagnosis, g*BRCA* status, clinical stage, and NACT regimen and pCR status. Analyses included all 293 patients. For one patient, only month and year of surgery were available; 15^th^ of the month was used. Assigning 1^st^ or 30^th^ of the month did not change the estimates.  * According to the 8th Edition of the Union for International Cancer Control (UICC) Tumor, Node, Metastasis (TNM) classification | | | | | | |
